# SARS-CoV-2 Antibody Prevalence Across Unvaccinated Health Care Workers During the COVID-19 Pandemic in Yemen: Cross-Sectional Study

**DOI:** 10.2196/67296

**Published:** 2025-08-29

**Authors:** Eihab Al-Herwi, Maryam Baras, Khadega Alhetar, Mohammed A Farhan, Rashad M Saleh, Amani As-suhbani, Manal Alshoaibi, Mansour Mohammed, Zainab Morshed, Asia Shujaa-Aldeen, Fatima Ghanem, Bra’ah AlQisi, Mohammed Alherwi, Tony Bruns

**Affiliations:** 1 Medical Laboratories Department, Faculty of Medicine and Health Sciences, Ibb University, University Street, Ibb, 22017, Yemen, 967 0778811234; 2 National Centre of Public Health Laboratories, Al-Mukalla, Yemen; 3 National Centre of Public Health Laboratories, Ibb, Yemen; 4 Medical Microbiology, Ibb University, Ibb, Yemen; 5 Medical Laboratories, International Malaysian University, Ibb, Yemen; 6 Pharmacy Department, University of Science and Technology, Ibb, Yemen; 7 Department of Internal Medicine III, University Hospital Rheinisch-Westfälische Technische Hochschule, Aachen, Germany

**Keywords:** SARS-CoV-2 antibodies, COVID-19, health care workers, risk factors, Yemen

## Abstract

**Background:**

The COVID-19 pandemic presented significant challenges to health care centers across Yemen. The lack of access to COVID-19 vaccines and limited availability of personal protective equipment greatly increased the risk of SARS-CoV-2 exposure among health care workers (HCWs). Only a few studies have examined the seroprevalence and burden of SARS-CoV-2 among Yemeni HCWs.

**Objective:**

This study aimed to assess the seroprevalence of SARS-CoV-2 and associated risk factors among a cohort of unvaccinated HCWs in Ibb City, the capital of Ibb Governorate, located in the highlands of southwestern Yemen between July 2022 and January 2023.

**Methods:**

Unvaccinated HCWs employed in public and private hospitals, dispensaries, pharmacies, and laboratories in Ibb City during the past 6 months were eligible. Blood samples, occupational information, and structured interviews using a questionnaire were collected from a convenience sample of 396 unvaccinated HCWs actively providing health care services between July 2022 and January 2023. SARS-CoV-2 antibody presence was determined using a lateral flow immunoassay.

**Results:**

Of the 396 HCWs tested, 268 (67.7%) were positive for SARS-CoV-2 antibodies, with no significant difference in seropositivity between sex (*P*=.29). Key factors associated with seropositivity included occupation and workplace. Compared to laboratory technicians (76/124, 61%), nurses (93/124, 75%; odds ratio [OR] 1.89, 95% CI 1.10‐3.26; *P*=.02) and physician assistants (13/14, 92.9%; OR 8.21, 95% CI 1.04‐64.79; *P*=.046) had significantly higher odds of seropositivity. Similarly, working in hospitals was associated with significantly higher odds of seropositivity compared to working in laboratories (OR 2.77, 95% CI 1.59‐4.81; *P*<.001). Overall, 82% (219/268) of seropositive HCWs reported COVID-19–related symptoms within the last 6 months (OR 3.82, 95% CI 2.40‐6.09; *P<.*001), the majority being fever (191/256, 74.6%; OR 2.40, 95% CI 1.56‐3.72; *P*<.001), headache (175/230, 76.1%; OR 2.50, 95% CI 1.62‐3.84; *P*<.001), cough (162/205, 79%; OR 3.02, 95% CI 1.94‐4.70; *P*<.001), or loss of taste (155/202, 76.7%; OR 2.36, 95% CI 1.53‐3.65; *P*<.001) or smell (146/191, 76.4%; OR 2.21, 95% CI 1.43‐3.41; *P*<.001).

**Conclusions:**

This study reveals a high prevalence of SARS-CoV-2 antibodies among HCWs in Ibb City, Yemen, underscoring the impact of limited vaccination and personal protective equipment availability in 2022 and 2023. These findings highlight the urgent need for improved protective measures and vaccination efforts in conflict-affected regions.

## Introduction

On March 11, 2020, the World Health Organization (WHO) officially declared the outbreak of COVID-19 a global pandemic [1]. Certain variants of SARS-CoV-2 have demonstrated enhanced replication efficiency, increased fitness, and heightened transmissibility, which may elevate the risk of reinfection [2]. As of August 4, 2024, the WHO reported more than 775 million confirmed cases of COVID-19 worldwide with more than 7 million related deaths [1]. The clinical spectrum of COVID-19 ranges from asymptomatic infection to severe viral pneumonia, acute respiratory distress syndrome, and death [2]. Identifying risk factors for SARS-CoV-2 infections [3] and for progression to severe disease or COVID-19–related hospitalization [4] is vital to guide prevention strategies and clinical management.

In conflict-affected and resource-poor countries like Yemen, where political instability and ongoing conflict have severely weakened the health care infrastructure, the pandemic has exacerbated existing challenges, with limited access to vaccines and personal protective equipment (PPE). Years of conflict with airstrike and bombardment since 2015 have left hospitals destroyed, equipment scarce, and health care workers (HCWs) struggling to provide care [5,6]. The WHO Regional Office for the Eastern Mediterranean reports fewer than 12,000 cumulative cases of COVID-19 until February 2024, with an estimated overall case fatality rate of 18.1% [7]. However, these numbers are likely to be inaccurate for several reasons, such as limited testing capacity and inconsistent data collection practices [8].

HCWs are positioned on the frontlines and face an elevated risk of contracting SARS-CoV-2 and potentially transmitting it to patients and others [9]. HCWs in conflict zones frequently operate in settings characterized by limited resources, insufficient PPE, and weakened health care infrastructure, contributing to elevated transmission rates [10]. In regions where the SARS-CoV-2 vaccine is unavailable and PPE supplies are limited, there are no clear data on the seroprevalence of the disease. As seroprevalence studies are difficult to carry out in war-torn nations, only a few studies have attempted to estimate the true burden of disease in Yemen [11,12], and only a few [13,14] have examined seroprevalence and COVID-19 burden among Yemeni HCWs.

Recognizing the limited data on SARS-CoV-2 seroprevalence among Yemeni HCWs, this study sought to determine the frequency of SARS-CoV-2 antibodies and investigate associated risk factors within an unvaccinated HCW cohort in Ibb, Yemen.

## Methods

### Patients and Setting

This cross-sectional study recruited 396 HCWs employed in public and private hospitals, dispensaries, pharmacies, and laboratories in Ibb City, the capital of Ibb Governorate located in the highlands of southwestern Yemen, between July 2022 and January 2023. A convenience sampling strategy was used for recruitment. Eligibility criteria for study inclusion required participants to be adults (age ≥18 years), employed in a health care setting for at least the 6 months preceding enrollment, unvaccinated against COVID-19, and to have provided written informed consent. The sole exclusion criterion was the previous receipt of 1 or more doses of any COVID-19 vaccine. To implement the convenience sampling, we visited various health care facilities during working hours. Eligible HCWs who were present, available, and willing to participate during the researchers’ visits were enrolled consecutively during the study period. For this, HCWs were visited at their workplaces during working hours and participated in structured interviews based on a predesigned questionnaire. Occupational and clinical data were collected from all participants during sample collection. HCWs were categorized according to the Yemen Medical Council’s classification, including physicians, nurses, dentists, pharmacists, physician assistants, and laboratory technicians.

### SARS-CoV-2 Antibody Testing

Capillary whole-blood samples were obtained from all participants, and SARS-CoV-2 IgM and IgG antibodies were detected using a lateral flow immunoassay test according to the manufacturer’s instructions (Clungene, Shanghai International Holding Corp). Briefly, 5 µL of whole blood and 80 µL of sample buffer were applied. If antibodies against SARS-CoV-2 were present, they bound to the labeled antigens, forming antigen-antibody complexes that migrated to the test lines, where immobilized capture antibodies specific for SARS-CoV-2 antibodies were located. Clinical symptoms within the past 6 months were recorded using a standardized questionnaire.

### Statistical Analysis

Data analysis was performed using SPSS (version 25.0; IBM Corp). Chi-square tests were used to assess associations between categorical variables. Logistic regression analyses were conducted to calculate odds ratios (ORs) and 95% CIs. The prevalence of positive test results was analyzed in relation to sex, age groups, occupation types, and symptoms. Statistical significance was defined as *P<*.05 for all tests without correction for multiple testing.

### Ethical Considerations

The study received ethical approval from the Research Ethics Committee of Ibb University, Faculty of Medicine and Health Sciences (registration number IBBUNI.AC.YEM. 2022.35). Informed consent was obtained from all participants. Before analysis, participants’ data were deidentified using pseudonymization. Participants were not financially compensated for participation in the study.

## Results

Between July 2022 and January 2023, capillary blood samples from 396 HCWs from Ibb City, Yemen, were collected. The mean age of enrolled participants was 28.6 (SD 7.8) years. The majority of HCWs in our study were nurses and laboratory technicians, accounting for 62.6% (248/396) of the participants (Table 1). None of the study participants were vaccinated.

**Table 1.** Demographic characteristics, occupation, and SARS-CoV-2 seropositivity among participating health care workers in Ibb City, Yemen (2022‐2023).

Characteristics	Number of examinees, n	Positive test, n (%)	Odds ratio (95% CI)	*P* value^a^
All participants	396	268 (67.7)		
Sex				
Female	207	145 (70)	Reference	
Male	189	123 (65.1)	0.80 (0.52‐1.22)	.29
Age groups (years)				
<30	264	176 (66.7)	Reference	
30‐39	92	59 (64.1)	0.89 (0.54‐1.47)	.66
40‐49	28	23 (82.1)	2.30 (0.85‐6.26)	.10
≥50	12	10 (83.3)	2.50 (0.54‐11.66)	.24
Occupation				
Laboratory technician	124	76 (61.3)	Reference	
Physician	66	46 (69.7)	1.45 (0.77‐2.75)	.25
Nurse	124	93 (75)	1.89 (1.10‐3.26)	.02
Dentist	27	21 (77.8)	2.21 (0.83‐5.87)	.11
Pharmacist	41	19 (46.3)	0.55 (0.27‐1.11)	.10
Physician assistant	14	13 (92.9)	8.21 (1.04‐64.79)	.046
Working place				
Laboratory	67	34 (50.7)	Reference	
Hospital	262	194 (74)	2.77 (1.59‐4.81)	<.001
Private clinic	29	18 (62.1)	1.59 (0.65‐3.87)	.31
Dispensary	16	10 (62.5)	1.62 (0.53‐4.96)	.84
Pharmacy	22	12 (54.5)	1.16 (0.44‐3.01)	.76
Symptoms^b^				
No	108	49 (45.4)	Reference	
Yes	288	219 (76)	3.82 (2.40‐6.09)	<.001

a *P* values were calculated by chi-square test.

b Within the past 6 months.

The overall seropositivity rate among HCWs was 67.7% (268/396), with no significant difference in seropositivity between sexes: 145 of 207 (70%) in females and 123 of 189 (65.1%) in males. HCWs aged 40 years or older (33/40, 82.5%) were associated with the higher odds of seropositivity (OR 2.42, 95% CI 1.04‐5.65) compared with those younger than 40 years (235/356, 66%; *P*=.03).

Compared with laboratory technicians as a reference group, the odds of being seropositive were significantly higher for physician assistants (OR 8.21, 95% CI 1.04‐64.79) and nurses (OR 1.89, 95% CI 1.10‐3.26). Dentists also showed higher odds of seropositivity (21/27, 77.8%; OR 2.21, 95% CI 0.83‐5.87), although this association was not statistically significant. Similarly, compared with working in laboratories, working in a hospital setting was associated with significantly higher odds of seropositivity (OR 2.77, 95% CI 1.59‐4.81; Table 1).

Among seropositive individuals, 81.7% (219/268) reported COVID-19–related symptoms within the last 6 months, the majority being fever (191/268, 71.2%), headache (175/268, 65.3%), cough (162/268, 60.4%), loss of taste (155/268, 57.8%), or smell (146/268, 54.4%; Table 2).

**Table 2.** Association between signs and symptoms and SARS-CoV-2 seropositivity among included health care workers in Ibb City, Yemen (2022‐2023).

Symptoms	Examinees, n	Positive test, n (%)	Odds ratio (95% CI)	*P* value^a^
Fever				
No	140	77 (55)	Reference	
Yes	256	191 (74.6)	2.40 (1.56‐3.72)	<.001
Chills				
No	233	143 (61.4)	Reference	
Yes	163	125 (76.7)	2.07 (1.32‐3.24)	.001
Cough				
No	191	106 (55.5)	Reference	
Yes	205	162 (79)	3.02 (1.94‐4.70)	<.001
Headache				
No	166	93 (56)	Reference	
Yes	230	175 (76.1)	2.50 (1.62‐3.84)	<.001
Breathing problem				
No	296	197 (66.6)	Reference	
Yes	100	71 (71)	1.23 (0.75‐2.02)	.41
Muscle pain				
No	230	145 (63)	Reference	
Yes	166	123 (74.1)	1.68 (1.08‐2.60)	.02
Loss of taste				
No	194	113 (58.2)	Reference	
Yes	202	155 (76.7)	2.36 (1.53‐3.65)	<.001
Loss of smell				
No	205	122 (59.5)	Reference	
Yes	191	146 (76.4)	2.21 (1.43‐3.41)	<.001
Sore throat				
No	262	162 (61.8)	Reference	
Yes	134	106 (79.1)	2.34 (1.44‐3.80)	<.001
Congestion or runny nose				
No	258	163 (63.2)	Reference	
Yes	138	105 (76.1)	1.85 (1.16‐2.96)	.009
Nausea or vomiting				
No	346	234 (67.6)	Reference	
Yes	50	34 (68.0)	1.02 (0.549‐1.92)	.96
Diarrhea				
No	354	237 (66.9)	Reference	
Yes	42	31 (73.8)	1.39 (0.68‐2.87)	.37

a The *P* values were calculated by chi-square test.

Having experienced symptoms within the past 6 months was associated with significantly higher odds of SARS-CoV-2 seropositivity (OR 3.82, 95% CI 2.40‐6.09). Specifically, participants who reported experiencing symptoms such as fever, chills, cough, headache, muscle aches, loss of taste, loss of smell, sore throat, and congestion (runny nose) had increased odds of being seropositive (Table 2). Among these, cough was associated with the highest odds of seropositivity (OR 3.021, 95% CI 1.94‐4.70; *P*<.001). In contrast, gastrointestinal symptoms, including nausea, vomiting, and diarrhea, did not demonstrate a positive association with SARS-CoV-2 seropositivity.

## Discussion

### Principal Findings

We herein report the serostatus for SARS-CoV-2 in 396 unvaccinated HCWs in Ibb City in 2022-2023 to be 67%. Seropositivity was highest in individuals working in a hospital (194/268, 74%) as compared with laboratory (34/268, 50.7%) or pharmacy staff (12/268, 54.5%).

Since the first laboratory-confirmed case of COVID-19 was reported in April 2020 in Mukalla City [15], Yemeni HCWs have faced the pandemic amid numerous challenges, including a scarcity of PPE, the absence of vaccines in many cities, ongoing conflict, and various crises affecting the country.

During the early stages of the pandemic, studies worldwide reported seropositivity rates in unvaccinated HCWs ranging from 8% to more than 40%, with large differences between countries and hospitals [16-18]. After the first wave of COVID-19 in Yemen, between May and September 2020, 19.4% of asymptomatic HCWs at the Médecins Sans Frontières Trauma Center in Aden, Yemen, tested seropositive for SARS-CoV-2 antibodies [13]. Several factors have contributed to the lack of COVID-19 vaccines in many regions of Yemen, including the ongoing conflict that has severely disrupted the health care system and damaged infrastructure, hindering vaccine delivery to remote or conflict-affected areas; limited resources; and misinformation or lack of awareness about the importance of vaccination. During the conflict, Ibb was deemed safer, attracting migrants and increasing population density. This influx strained limited health care services, complicating COVID-19 control efforts.

From late 2022 to early 2023, many countries, including those in the Arab world, began to consider COVID-19 an endemic disease as infection rates stabilized and COVID-19 cases became more predictable with fewer severe outcomes [19]. The case fatality rate of confirmed infections gradually decreased in Taiz Governorate in southern Yemen from 27% in 2020 to 12.8% and 5.8% in 2021 and 2022, respectively [12]. In parallel, the seroprevalence of SARS-CoV-2 among HCWs from 9 hospitals in the Lahj and Al-Dhalea governorates of Yemen increased to 94.2% between June 2022 and September 2022, underscoring the transition to an endemic situation [14]. Consistent with our findings, a meta-analysis of COVID-19 seroprevalence conducted across 49 studies involving 127,480 HCWs indicated that antibody prevalence varied from 0% to 45.3%. Notably, one of the key factors associated with higher seroprevalence rates was the shortage of PPE [14]. In line with our study, the war-torn conditions and limited availability of PPE had a significant impact on the increase in antibody prevalence (56.5%) of COVID-19 among HCWs in Tigray, Ethiopia [14]. Although this study also included several professions, such as nurses, radiologists, dentists, laboratory personnel, pharmacists, respiratory therapists, and nutritionists, we here report a considerably lower seroprevalence in a comparable time frame with significant occupation-related differences. In our study, direct patient care as a physician assistant, dentist, nurse, or physician was associated with the highest odds of seropositivity.

Our findings align with other studies that identified higher odds of infection or seropositivity among health care staff who provided direct patient care in confined spaces, had prolonged or repeated face-to-face contact, or had contact with the environment or materials used by patients with COVID-19, which presumably contributed to the increased risk in these occupational groups [20-22]. The inadequate availability of PPE in hospitals may also have contributed to this risk, given the higher number of HCWs in hospitals compared with laboratories. Other studies [23-25] have found no significant correlation between occupation and infection, possibly reflecting differences in the availability and use of PPE and possible exposure from colleagues and household members.

In our study, seroprevalence of SARS-CoV-2 was highest among HCWs aged 40 years or older (33/40, 82.5%), compared with those younger than 40 years (235/356, 66%). This contrasts with studies from the Western countries, where higher seropositivity rates have been reported among younger HCWs, possibly due to increased exposure through social behaviors and patient contact, as well as differences in adherence to stringent protective measures [16,26].

Our analysis indicated significantly higher odds of SARS-CoV-2 seropositivity associated with typical symptoms such as fever, chills, headache, loss of taste, loss of smell, and sore throat. Other studies in HCWs have highlighted a strong association of loss of taste or smell with seropositivity [27-29]. In our study, loss of taste or smell was observed in 57.8% (155/268) of seropositive HCWs and associated with 2.3-fold odds. Notably, anosmia within the last 6 months was also reported by 45 (35%) seronegative HCWs, which is considerably higher than the self-reported olfactory loss (<15%) or objective smell dysfunction (<25%) in the general population [30]. This might, in part, be explained by false-negative results from the assays used, which represents a limitation of the study.

Several limitations warrant discussion. First, the recruitment strategy used may have introduced selection bias, potentially limiting the generalizability of our findings. Although a large sample size of 396 HCWs was enrolled, this sample may not be fully representative of all unvaccinated HCWs in Ibb, Yemen. Furthermore, the composition of the study sample might not accurately reflect the overall workforce in the specific health care facilities from which participants were drawn. This may affect the generalizability of the results to other regions or health care settings. In addition, this study relied on structured interviews and self-reported data for occupational information and COVID-19–related symptoms, which may have introduced recall bias or inaccuracies in reporting.

In summary, the observed seroprevalence of SARS-CoV-2 among HCWs in Ibb City, Yemen, highlights the ongoing vulnerability of HCWs, particularly those with close patient contact, in resource-limited and conflict-affected regions. These findings underscore the urgent need for sustained efforts to enhance infection control measures, including the provision of PPE and broader access to vaccines. Monitoring occupation-related risks and the evolving epidemiological landscape is crucial for informing future public health strategies.

### Conclusions

This study reveals a high prevalence of SARS-CoV-2 antibodies among unvaccinated HCWs in Ibb City, indicating significant exposure to the virus during the pandemic. The analysis identified key risk factors, particularly occupation and workplace settings, with nurses and physician assistants having elevated odds of infection. These findings highlight the urgent need for targeted interventions to protect HCWs, including enhanced safety protocols and vaccination campaigns. Furthermore, the data suggest the necessity for ongoing surveillance and support for HCWs, especially in high-risk environments such as hospitals, to mitigate the spread of COVID-19 and ensure the safety of both health care providers and patients. Continued research is essential to understand the long-term implications of COVID-19 exposure among HCWs and to develop targeted interventions that can more effectively protect this critical workforce in similar settings worldwide.

## Abbreviations

HCW: health care worker
OR: odds ratio
PPE: personal protective equipment
WHO: World Health Organization
